# The efficacy of different doses of citicoline in improving the prognosis of patients with acute ischemic stroke based on network meta-analysis

**DOI:** 10.3389/fphar.2025.1529647

**Published:** 2025-04-04

**Authors:** Xu Zhao, Xianhao Huo, Yizhen Meng, Ran Zhao, Xiaozhuo Liu, Jiancheng Chen, Zhiqi Mao, Mei Li

**Affiliations:** ^1^ Department of Neurosurgery, North China University of Science and Technology Affiliated Hospital, Tangshan, Hebei, China; ^2^ Department of Neurosurgery, General Hospital of Ningxia Medical University, Yinchuan, China; ^3^ Department of Ultrasound, North China University of Science and Technology Affiliated Hospital, Tangshan, Hebei, China; ^4^ Department of Neurology, The Second Affiliated Hospital, Hengyang Medical School, University of South China, Hengyang, Hunan, China; ^5^ Department of Neurosurgery, First Medical Center, Chinese PLA General Hospital, Beijing, China

**Keywords:** ischemic stroke, citicoline, neurological function, daily living activities, network meta-analysis

## Abstract

**Objective:**

Our group aimed to explore the effect of different dosages of citicoline on ischemic stroke (IS) patients and determine the most appropriate dosage for these patients.

**Methods:**

The databases of PubMed, Cochrane Library, Medline, Web of Science, and Embase were searched from their establishment to 15 October 2024. We assessed the quality of all included articles by using the Cochrane quality evaluation method or Newcastle-Ottawa Scale (NOS), which was based on the study type. Relative risk (RR) and 95% confidence interval (CI) were used for dichotomous data, and mean and standardized difference (SD) were used for continuous data. The outcome indicators were death, improvement in neurological function and daily living activities, and adverse effects.

**Results:**

In this study, a total of 13 studies were included. Of these, 370 patients were treated with 500 mg citicoline, 502 patients were treated with 1,000 mg citicoline, 1,891 patients were treated with 2,000 mg citicoline, and 2,582 patients were treated in the group of control (CON). We evaluated the treatment effect of different outcome indicators by ranking. In terms of death, both 500 mg citicoline and 2,000 mg citicoline demonstrated lower mortality than CON, with 2,000 mg citicoline having the lowest mortality. In terms of neurological function improvement, we found that compared to CON, the rates of improvement were higher and the rates of ineffective results were lower in 500-mg citicoline, 2,000-mg citicoline, and 1,000-mg citicoline groups. In terms of improvement in daily living activities, the MBI scores for 500 mg citicoline and 2000 mg citicoline were both higher than CON, while the MBI score for 1,000 mg citicoline was not. Lastly, in the aspect of adverse effects, we found that the rate of adverse effects was lower for 1,000 mg citicoline than CON, while it was higher for 500 mg citicoline and 2,000 mg citicoline.

**Conclusion:**

Our research findings revealed that different dosages of citicoline significantly affect the neurological function, daily living activities, and adverse effects in patients with acute IS. Notably, 500 mg citicoline and 2,000 mg citicoline not only demonstrate higher rates of improvement in neurological function and daily living activities but also have lower mortality and ineffective results. However, this study does not specify the best one of the two dosages.

## Background

Ischemic stroke (IS) is the leading cause of long-term disability and death (Ouyang et al., 2025). More than 50% of stroke survivors present persistent disability, and about 30% have partial dependence in activities of daily living 6 months after stroke (Premi et al., 2022). Currently, the therapeutic approaches for IS mainly involved two strategies: first, the restoration of blood flow by thrombolysis or mechanical thrombectomy within the initial hours of IS occurrence, which is considered one of the most effective interventions, leading to improved functional recovery and clinical outcomes; second, neuroprotective strategies, which may be applicable to a broader spectrum of IS patients (Ghannam et al., 2023; Jadhav et al., 2021; Martynov and Gusev, 2015).

Citicoline, known as cytidine-5′-diphosphocholine (CDP-choline), is metabolized into cytidine and choline through hydrolysis and dephosphorylation processes in the human body (Jasielski et al., 2020). These two substances serve as key substrates for neurons to synthesize phosphatidylcholine and cytidine-5′-diphosphocholine (Prinz et al., 2023). As a multimodal drug, CDP-choline exhibits comprehensive neuroprotective effects and has demonstrated neuroprotection and neurogenesis in various central nervous system experimental and clinical conditions, including acute and chronic IS, intracranial hemorrhage (ICH), Parkinson’s disease, and Alzheimer’s disease; citicoline is also beneficial in glaucoma and amblyopia (An et al., 2020; Castagna et al., 2021; Cavalu et al., 2024; Iulia et al., 2017; Sbardella et al., 2020). CDP stands out as the sole medication that has consistently demonstrated neuroprotective effects across a variety of stroke clinical trials (Agarwal et al., 2022; Hurtado et al., 2011; Li et al., 2023). This drug is not only safe and well-tolerated but also holds broad therapeutic potential. Its neuroprotective properties have been scientifically established (Bermejo et al., 2023; Overgaard, 2014). However, the effect of different doses of citicoline on the prognosis of patients with traumatic brain injury (TBI) has been explored only in a meta-analysis conducted by Secades et al. (2023) in the moment and found that there was no effect of the different doses on the prognosis of TBI-related patients. Moreover, we also know that the administration dosages of citicoline exhibit diversity in IS-related clinical trials, with the primarily dosages being 500 mg, 1,000 mg, and 2,000 mg (Alvarez-Sabín et al., 2016; Dávalos et al., 2012; Warach et al., 2000). Currently, the studies of citicoline primarily focus on the impact of citicoline compared to placebo or other neuroprotective drugs on the patients of IS, with little exploration into whether different dosages of citicoline have a specific effect on the prognosis of these patients.

The network meta-analysis is a technique that integrates direct and indirect evidence from a network of randomized controlled trials to simultaneously compare multiple interventions within a single analytical framework (Rouse et al., 2017). It is also helpful in evaluating the comparative effectiveness of different interventions (Nino and Brignardello-Petersen, 2023). Therefore, our group aimed to conduct a comprehensive search of all clinical trials related to the use of citicoline for improving the prognosis of patients with acute IS (by using the control group as a reference), explore the effect of different dosages of citicoline on IS patients, and determine the most appropriate dosage for these patients.

## Patients and methods

Our network meta-analysis was performed according to the checklist of the Preferred Reporting Items for Systematic reviews and Meta-Analyses (PRISMA) extension statement (Hutton et al., 2015).

### Search strategy and data collection

The databases of PubMed, Cochrane Library, Medline, Web of Science, and Embase were searched from their establishment to 15 October 2024. The retrieval formula was ((((((ischemic stroke) OR (stroke)) OR (brain stroke)) OR (cerebral stroke)) OR (brain ischemic stroke)) OR (cerebral ischemic stroke)) AND (((citicoline) OR (CDP)). Meanwhile, we also manually searched the original research, which were included in the published of relevant meta-analysis and systematic review, ongoing or completed unpublished trials, and abstracts.

There were two reviewers to screen all articles independently. They also extracted the data from all included studies and information as follows: the first author or corresponding author, study type, publication of year and country, number of patients, interventions, details of interventions, and the outcome indicators. If data were missing, we contacted the authors of the study.

### Eligibility criteria

The search strategy was based on the PICOS principle (P: population/patient, I: intervention, C: control/comparison, O: outcome, S: study design) (Lu et al., 2023). In terms of patients, the following criteria were included: the patients who experienced ischemic stroke for the first time and those aged 18 or older, the NIHSS score ≥3, and the time from stroke onset to begin treatment was ≤72 h. In terms of interventions, the following criteria were considered: the group administered with citicoline was considered the treatment group, and the control group was sham. In terms of study design, the following criteria were considered: we included both randomized controlled clinical trials and non-randomized controlled trials. In terms of outcomes, the following criteria were considered: the outcome indicators are mortality, the rate of favorable effect (it was evaluated by the modified Rankin Scale score (mRS) or National Institutes of Health Stroke Scale (NIHSS) score, which were used to evaluate the severity of neurological deficits, and the higher score indicates a more severe degree of neurological deficits), the activities of daily living (ADLs) (it was evaluated by using the modified Barthel index (MBI), which was an effective, reliable, and sensitive tool for evaluating the activities of daily living in the aspects of feeding, dressing, toileting, transferring, ambulation, and stair climbing with patients experiencing stroke; the higher the score, the better the ability of daily living), and adverse effects.

The exclusion criteria included the following: studies including patients with other brain injury (e.g., hemorrhagic stroke and traumatic brain injury), studies including patients who had severe complications and could not tolerate treatment (like heart, liver, or renal dysfunction), single case reports, single-arm trials, studies without the outcome indicators, animal experiments, and reviews.

### The risk of bias

Two reviewers assessed the quality of included articles. All randomized clinical trials (RCTs) were assessed by using the Cochrane quality evaluation method from six aspects (Tan et al., 2023); all of these has three levels and are represented by three colors (green for low risk of bias, yellow for unclear risk of bias, and red for high risk of bias). The non-randomized clinical trials were assessed by using the Newcastle-Ottawa Scale (NOS) from three aspects (Lin et al., 2020), with the score ≥5 indicating high quality of these articles.

### Data analysis

In this study, the relative risk (RR) and 95% confidence interval (CI) were used for dichotomous data, and mean and standardized difference (SD) were used for continuous data. The heterogeneous test, transitivity, inconsistency test, and publication bias were conducted for all included trials. In terms of heterogeneous, there was non-heterogeneity with P > 0.1 and I^2^<50%, and the fixed model was adopted; otherwise, a random-effects model was applied. In terms of transitivity, the clinical and methodological variables (e.g., age, sex, and the time from stroke onset to initiation of treatment) were compared between the different interventions. In terms of inconsistency, it was assessed by using the node-splitting method between the direct and indirect evidence. Last, we ranked the treatment effect of all interventions by using the surface under the cumulative ranking curve (SUCRA). The traditional meta-analysis was performed using RevMan 5.3 (Cochrane Collaboration, London, United Kingdom), and the network meta-analysis was performed using Stata 16.0 (StataCorp, TX, United States). Image processing involved in this study was completed using Adobe Illustrator 2021 (Adobe Systems Inc., San Jose, CA, United States).

## Results

### Search results and study characteristics

**FIGURE 1:**
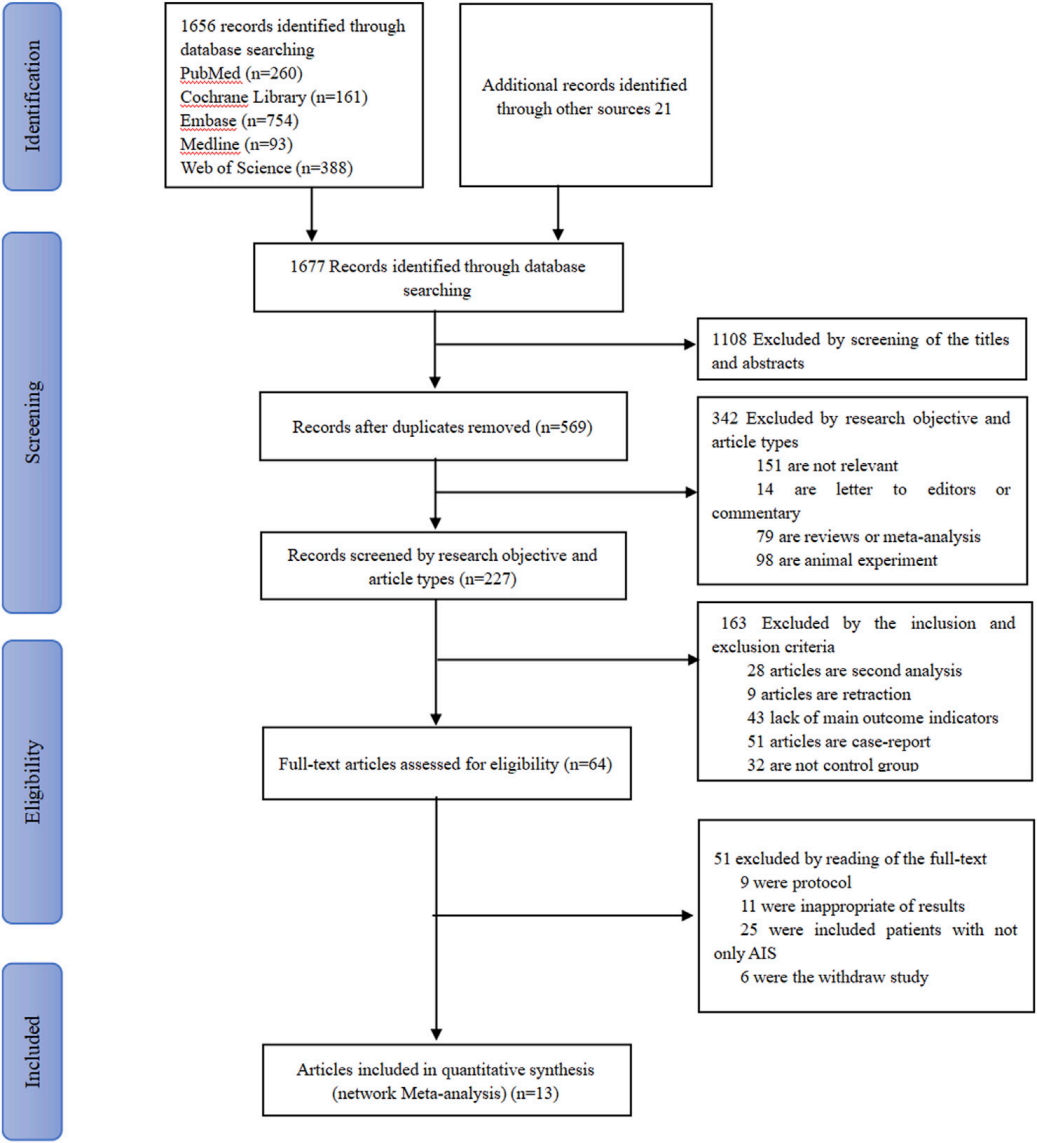
Flowchart of the study selection process.

A total of 1,677 studies were retrieved. First, 1108 duplicate studies were removed by reading titles and abstracts. Then, 569 studies were screened by reading the research objective and article type, as a result of which 342 studies were excluded (the reasons were that not relevant, letter to editors or commentary, reviews, and animal experiments). In addition, based on inclusion and exclusion, we screened 227 studies and excluded 163 of them because of the retraction of articles, lack of main outcome indicators, single-arm study, and case report. Finally, after 51 articles were excluded (due to protocols, included patients with TBI/ICH, etc.), the remaining 13 articles were included for network meta-analysis, which included 10 randomized control trials (Agarwal et al., 2022; Alvarez-Sabín et al., 2013; Alvarez-Sabín et al., 2016; Clark et al., 2001; Clark et al., 1997; Clark et al., 1999; Dávalos et al., 2012; Mitta et al., 2012; Tazaki et al., 1988; Warach et al., 2000), 2 retrospective studies (Leon-Jimenez et al., 2010), and 1 prospective study (Mehta et al., 2019). The screening flowchart is shown in Figure 1. Of these, 370 patients were treated with 500 mg citicoline, 502 patients were treated with 1,000 mg citicoline, 1,891 patients were treated with 2,000 mg citicoline, and 2,582 patients were treated in the group of Control (CON) (in this group, the patients were not treated with citicoline or other neuroprotective drugs but were only treated with anti-hypertensive, lipid-lowering, anticoagulant, etc.). Geographically, 5 (38.5%) studies were conducted in United States, 3 (23.1%) in India, 2 (15.4%) in Spain, 1 (7.7%) in Japan, 1 (7.7%) in Russia, and 1 (7.7) in Mexico (Supplementary Table S1).

### Risk of bias

**FIGURE 2:**
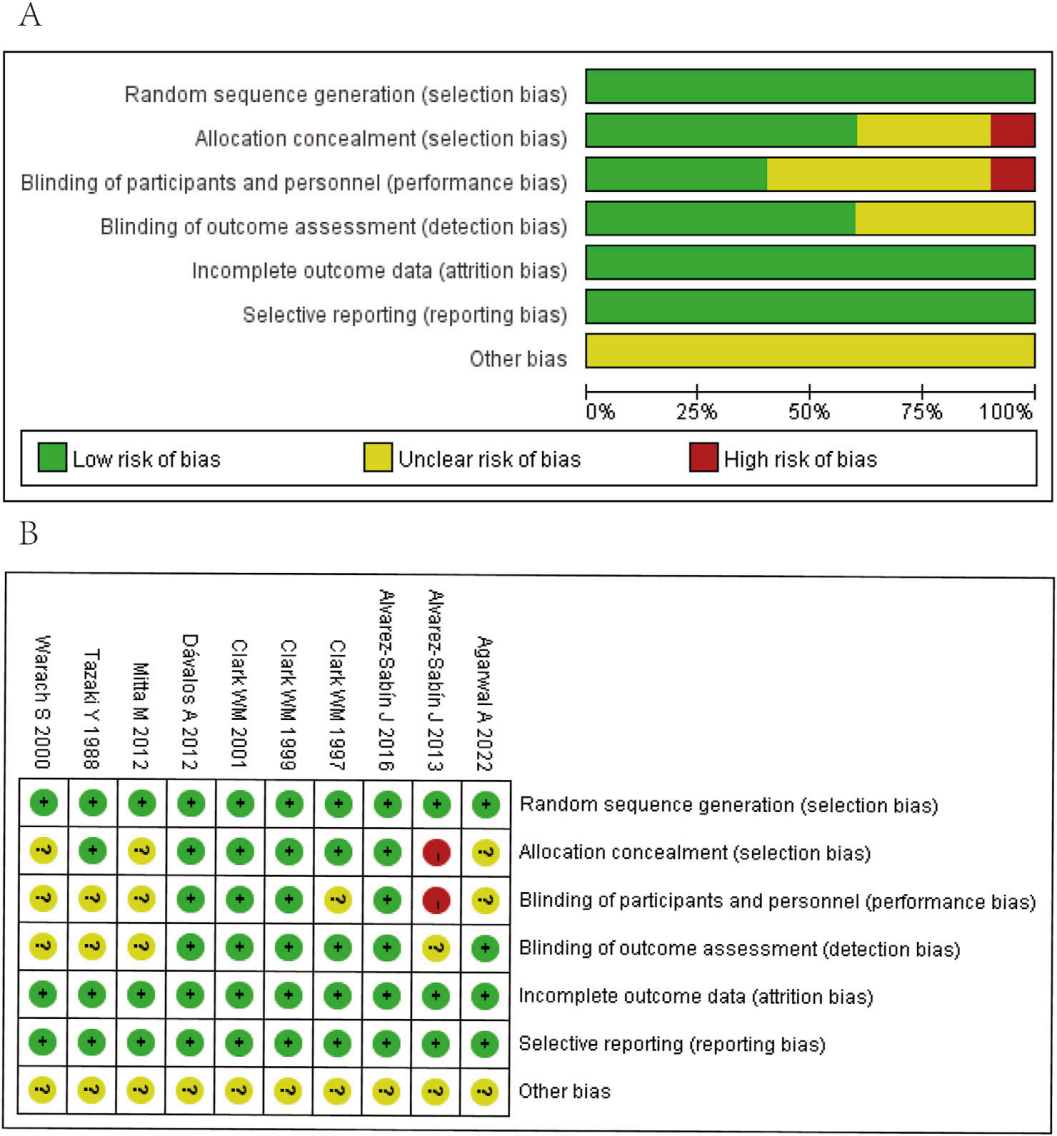
Quality assessment of identified randomized controlled trials. **(A)** Each risk of bias item presented as percentages across all included studies. **(B)** Each risk of bias item for each included study. Green indicates a low risk of bias, yellow indicates an unclear risk of bias, and red indicates a high risk of bias.

The included 10 RCTs were assessed by using the Cochrane risk of bias tool, and all of them employed the correct randomization methods and were free from attrition bias and reporting bias. Regarding other bias risks, the study by Alvarez-Sabín et al. (2013) did not implement adequate allocation concealment and blinding of participants and personnel; the studies by Mitta et al. (2012), Alvarez-Sabín et al. (2016), Tazaki et al. (1988) and Warach et al. (2000) were unclear whether the correct allocation concealment and blinding of participants and personnel were used; Alvarez-Sabín et al. (2013), Mitta et al. (2012) Tazaki et al. (1988), and Warach et al. (2000) were also unclear whether the correct blinding of outcome assessment was used. Therefore, we consider that the quality of all RCTs included in our analysis was moderate (Figures 2A, B). The three other non-RCTs were analyzed by the NOS assessment tool, which assigned high scores in the aspects of selectivity, comparability, and outcome assessment. Thus, we assume that the quality of all non-RCTs was high (Table 1).

**TABLE 1:** Quality assessment of the non-RCT studies.

Study	Selection	Comparability	Outcome	Total score
Martynov Mlu	4	2	2	8
Leon-Jimenez C	4	2	3	9
Mehta A	4	1	3	9

A score of 5 or less indicates a high risk of bias.

We also assessed the publication bias of all included articles and found no publication bias in the aspects of mortality, ineffective results, and MBI (Supplementary Figure S1). However, there had been publication bias in the aspects of effective results and adverse effects. Specifically, the publication bias in effective result may primarily be attributed to the study by Alvarez-Sabín et al. (2016), while that in the aspect of adverse effect may be associated with the study by Miu et al. (2012) (Supplementary Figure S2).

### Assessing heterogeneity, transitivity, and inconsistency

For all included studies, we conducted a heterogeneity test in the traditional meta-analysis and found that there was no significant heterogeneity in direct comparisons regarding the death (all *I* ^*2*^ > 50%, *P* > 0.1). However, the heterogeneity was observed in direct comparison between 1,000 mg citicoline and CON in terms of favorable result and adverse effect, likely due to differences in the study design between the study by Alvarez-Sabín et al. (2016) and other studies within this subgroup. In the aspect of ineffective result, heterogeneity was noted in the direct comparison between 2,000 mg citicoline and CON, possibly attributed to differences in the study design between the study Miu et al. (2012) and other studies in this subgroup. Regarding MBI, heterogeneity stemmed from the direct comparison between 500 mg citicoline and CON, potentially due to variations in the study design (Tables 2, 3). In transitivity, we found that most comparisons differed in baseline NIHSS score, mean age, and the number of male patients (Supplementary Figure S3). In the test of inconsistency, we found that there was no evidence of inconsistency in the aspects of death, favorable results, ineffective results, MBI, and adverse effects (all *P* > 0.05; Supplementary Tables S2–4).

**TABLE 2:** Heterogeneity test of the traditional meta-analysis.

	Death			Favorable result			Ineffective result			MBI			Adverse effect		
	N	*P*	*I* ^*2*^	N	*P*	*I* ^*2*^	N	*P*	*I* ^*2*^	N	*P*	*I* ^*2*^	*N*	*P*	*I* ^*2*^
500 mg citicoline vs. CON	3	0.33	9%	3	0.15	47%	2	0.38	0%	2	0.05	75%	2	0.53	0%
1,000 mg citicoline vs. CON	5	0.52	0%	5	0.02	67%	3	0.80	0%	NR	NR	NR	3	0.04	77%
2,000 mg citicoline vs. CON	6	0.62	0%	6	0.14	40%	3	0.04	68%	4	0.19	37%	2	0.36	0%

^a^ N, number of studies; NA, not available.

**TABLE 3:** Analysis of traditional meta-analysis for direct comparisons.

	Death RR (95% CI); *P*	Favorable result RR (95% CI) *P*	Ineffective effect RR (95% CI) *P*	MBI RR (95%CI) *P*	Adverse effect RR (95%CI) *P*
500 mg citicoline vs. CON	0.99 [0.68, 1.45] *P = 0.97*	1.19 [0.90, 1.58] *P* = 0.23	0.92 [0.52, 1.63] *P* = 0.78	1.25 [0.76, 2.03] *P* = 0.38	1.79 [1.04, 3.08] *P* = 0.04
1,000 mg citicoline vs. CON	1.01 [0.67, 1.53] *P* = 0.96	1.40 [1.08, 1.81] *P* = 0.01	0.71 [0.51, 0.98] *P* = 0.04	0.89 [0.53, 1.50] *P* = 0.66	1.08 [0.86, 1.34] *P* = 0.52
2,000 mg citicoline vs. CON	0.90 [0.79, 1.03] *P* = 0.13	1.06 [0.94, 1.18] *P = 0.33*	0.81 [0.45, 1.45] *P* = 0.48	1.06 [0.91, 1.24] *P* = 0.44	1.10 [0.70, 1.75] *P* = 0.67

### The results of the network meta-analysis

**FIGURE 3:**
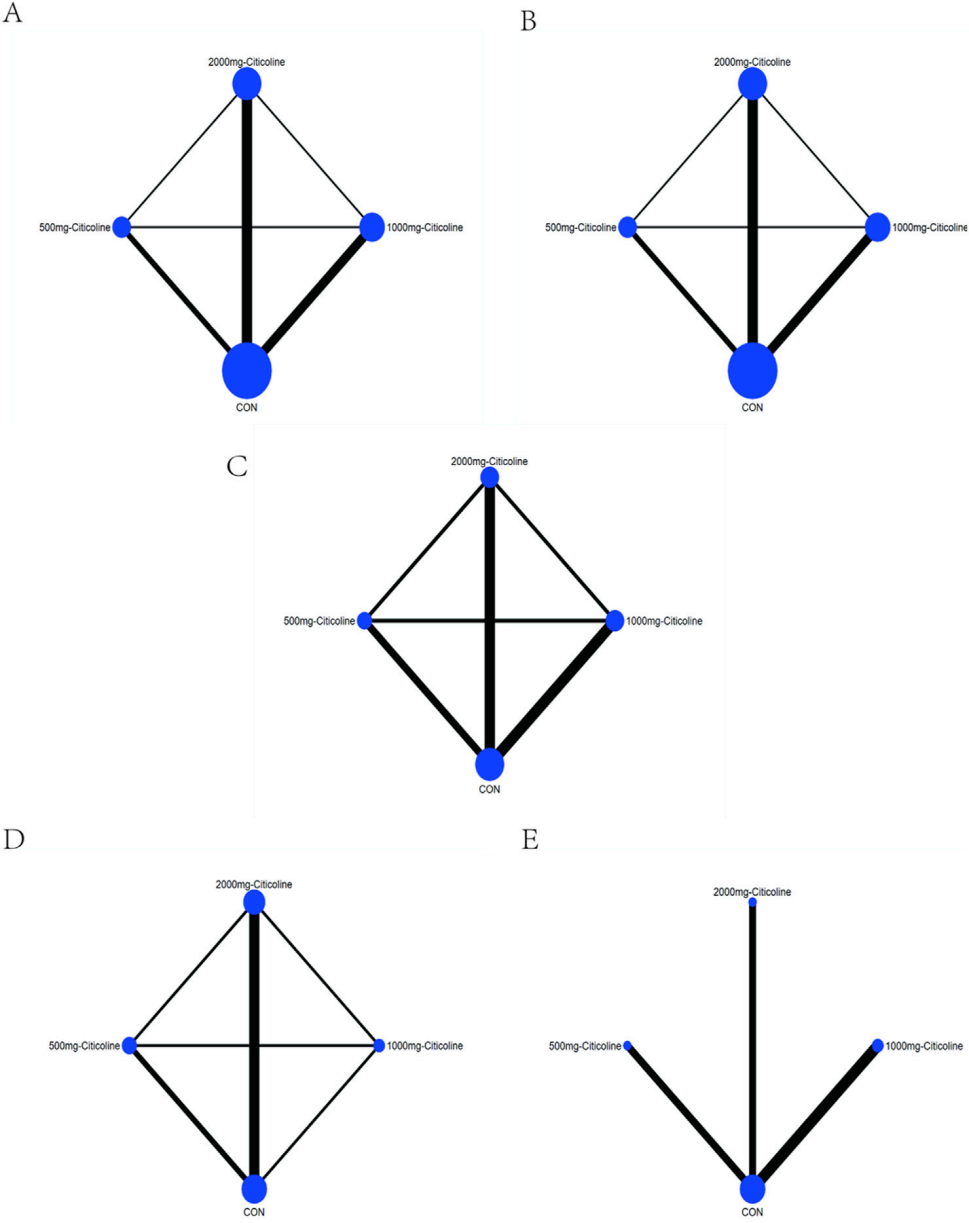
Map of network. **(A)** Network map based on the death of AIS. **(B)** Network map based on the patient proportion of the favorable result of AIS. **(C)** Network map based on the patient proportion of the ineffective rate of AIS. **(D)** Network map based on the patient proportion of the activities of daily living of AIS. **(E)** Network map based on the patient proportion of the adverse effect of AIS.

The line between two interventions indicated there is direct comparison evidence and vice versa. The size of the dots represents the sample size, and the thickness of lines represents the number of studies. In our study, we can obtain some information from the map of network (Figure 3). Taking Figure 3A as an example, we observe that there were three different doses of citicoline, which are 500 mg, 1,000 mg, and 2,000 mg, respectively. The greatest number of original studies with direct comparative evidence is between 2,000 mg citicoline and CON, followed by 1,000 mg citicoline and CON and then 500 mg citicoline and CON. In terms of sample size, the intervention with the largest sample size is CON, followed by 2,000 mg citicoline, 1,000 mg citicoline, and finally 500 mg citicoline.

**FIGURE 4:**
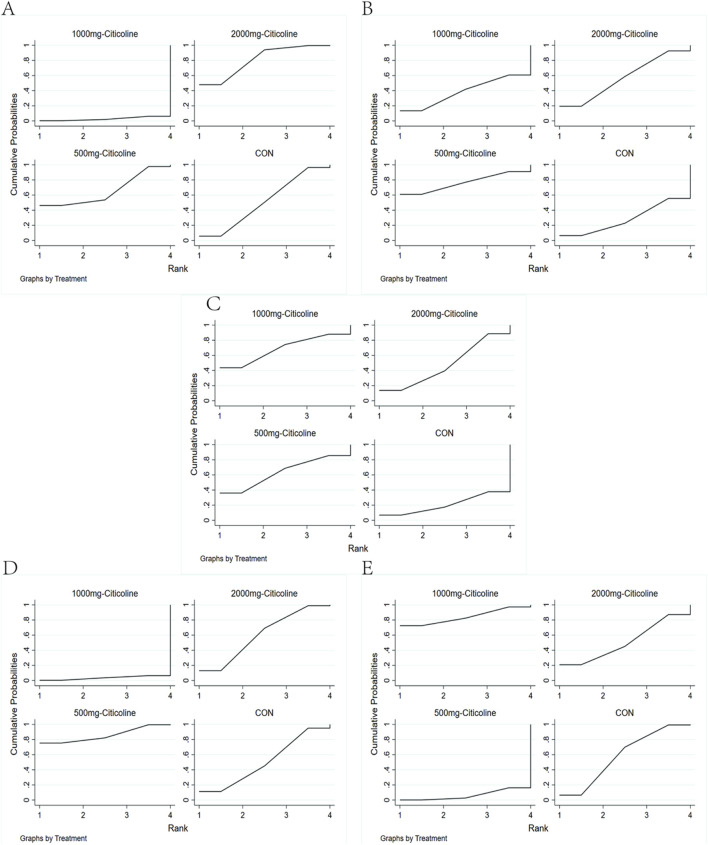
Rank chart. **(A)** Rank chart based on the death of AIS. **(B)** Rank chart based on the patient proportion of the favorable result of AIS. **(C)** Rank chart based on the patient proportion of the ineffective rate of AIS. **(D)** Rank chart base on the proportion of the activities of daily living. **(E)** Rank chart based on the patient proportion of the adverse effect of AIS.

In this study, we employed SUCRA to evaluate and rank the treatment effect of different outcome indicators. In the aspects of death, ineffective results, and adverse effect, the larger area under the curve corresponds to a lower rate of these outcomes. Conversely, in the aspects of favorable results and MBI, the larger area under the curve signifies a greater degree of improvement in patients. Among them, in the aspect of death (Figure 4A), our analysis revealed, compared to the CON, both 500 mg citicoline and 2,000 mg citicoline demonstrated lower mortality, with 2,000 mg citicoline having the lowest mortality. However, compared to the CON, 1,000 mg citicoline had a higher mortality, and the rank from the lowest to highest was 2,000 mg citicoline, 500 mg citicoline, CON, and 1000 mg citicoline.

In the aspect of neurological function improvement, we found that compared to the CON, the rates of improvement were higher and the rates of ineffective result were lower in the 500-mg citicoline, 2,000-mg citicoline, and 1,000-mg citicoline groups. Regarding favorable result (Figure 4B), the ranking from the highest to lowest was 500 mg citicoline, 2,000 mg citicoline, 1,000 mg citicoline, and CON. In terms of ineffective result (Figure 4C), the ranking from the lowest to highest was 1,000 mg citicoline, 500 mg citicoline, 2,000 mg citicoline, and CON.

In terms of improvement in activities of daily living (Figure 4D), the MBI scores for 500 mg citicoline and 2,000 mg citicoline were both higher than the CON, while the MBI score for 1,000 mg citicoline was not, with the ranking from highest to lowest being 500 mg citicoline, 2,000 mg citicoline, CON, and 1,000 mg citicoline.

Lastly, in the aspect of adverse effect (Figure 4E), we found that compared to the CON, the rate of adverse effect was lower for 1,000 mg citicoline, while it was higher for 500 mg citicoline and 2,000 mg citicoline; the ranking from the lowest to highest was 1,000 mg citicoline, CON, 500 mg citicoline, and 2,000 mg citicoline.

## Discussion

Citicoline is a naturally occurring compound present in all human cells, serving not only as an endogenous substance but also exhibiting neuroprotective properties (Cavalu et al., 2024). To date, it has been extensively studied in patients with various neurological disorders (Gareri et al., 2024; Grgac et al., 2024). However, in patients with IS, the efficacy of citicoline has yielded contradictory results. Some studies support the beneficial effects of it on the clinical indicators; a large RCT conducted by Dávalos et al. (2012) found no significant differences between citicoline and CON in terms of neurological improvement and the incidence of adverse effect. Moreover, our group conducted a network meta-analysis to explore the efficacy of different neuroprotective drugs in patients with acute IS recently (Li et al., 2024). Furthermore, it revealed that compared to CON, citicoline demonstrated a higher rate of neurological improvement, as well as lower rates of ineffective result and mortality. Therefore, we posit that citicoline may serve as an effective neuroprotective drug for patients with IS. However, in this analysis, we observed variations in the dosages of citicoline used among different studies. Consequently, we pose two critical questions: (1) Does the varying dosages of citicoline affect the prognosis of patients with IS? (2) Is the contradictory finding regarding this drug in these patients related to the differing dosages employed across different studies?

Based on this, we conducted a network meta-analysis to explore the effect of different dosages with citicoline on the prognosis of patients with IS. We found that both 500 mg citicoline and 2,000 mg citicoline not only had higher rates of improvement in neurological function and activities of daily living but also had lower mortality and lower ineffective result. This is the first study in nearly 30 years to explore the effect of different doses of citicoline on prognosis of patients with acute IS since the study by Clark et al. (1997). Similar to our findings, their results show that 500 mg and 2,000 mg doses of citicoline are associated with a better improvement rate of the activities of daily living, with the optimal dose identified as 500 mg.

However, by ranking, we found that 500 mg citicoline was the most effective in improving neurological function and activities of daily outcomes and 2,000 mg citicoline was the most effective in reducing mortality. Moreover, both 500 mg citicoline and 2,000 mg citicoline showed a higher rate of adverse effect, with the worst being 500 mg citicoline. Therefore, out of caution, we did not specify which dose was the most effective. On the other hand, the number of original studies and sample size for 500 mg citicoline were both smaller than those for 2,000 mg citicoline, which is another reason why we did not determine the optimal dose. In particular, in the terms of adverse effect, both 500 mg citicoline and 2,000 mg citicoline were only involved in two original studies. Thus, we believe that still, large-scale, high-quality RCTs are needed to further verify the efficacy and safety of these two doses in treating patients with acute IS, in hopes of determining the optimal dose in the future. Moreover, given the absence of direct data from subgroups with varying severity levels in the original studies, stratified analysis based on severity was not feasible in the analysis. Consequently, it remains undetermined which doses would be more effective for severe cases. This limitation is also needed to be addressed in future research.

In the dosage of 1,000 mg citicoline, we found that compared to the CON, it is associated with a better rate of neurological improvement and a lower rate of ineffective result but also with a higher mortality and a lower capacity for activities of daily living. This finding is similar to the conclusions conducted by Tazaki et al. (1988) who also noted that citicoline had a better rate of neurological improvement compared to the placebo. However, this contrasts with the findings of Agarwal et al. (2022) whose study revealed that there were no significant differences between citicoline and placebo in terms of improving neurological function and activities of daily living. Furthermore, although the incidence of adverse effect at this dosage was the lowest, only three studies with a small sample size were involved. Additionally, in terms of improvement in neurological function/daily living activities and reduction in mortality, this dosage was not superior to 500 mg citicoline and 2,000 mg citicoline. The conclusion regarding the improvement in daily living activities was derived from indirect comparative evidence; there is currently no direct comparative evidence to explore the effect of this dose on the daily activities of these patients. Consequently, after a comprehensive consideration, we deem it necessary to conduct a renewed and thorough evaluation of the therapeutic effect of the 1,000 mg citicoline dosage in these patients.

There are several limitations in this analysis. First, the analysis did not explore whether the different time frames of citicoline administration had an effect on outcomes; therefore, it cannot infer that these doses may be optimal for a narrower window, which will require further investigation in the future. Second, only one large RCT has explored the therapeutic effects of 2,000 mg citicoline in patients with acute IS; the number of original studies and sample size for 500 mg citicoline and 1,000 mg citicoline are both small, which may reduce the strength of evidence from this study. Consequently, to further substantiate these findings, it is necessary to augment the sample size and conduct additional studies. Moreover, to date, only one randomized controlled clinical trial conducted in 1997 has directly compared the impact of these three dosages of citicoline in patients with acute IS. Currently, direct comparative evidence regarding these three dosages remains scarce, necessitating additional direct comparative evidence to further validate their therapeutic effects in these patients and substantiate the conclusions drawn from this study. Ultimately, we found that there are different routes of administration of citicoline and doubt these may influence the therapeutic outcomes. Specifically, in this analysis, 500 mg citicoline was exclusively administered orally, while 1000 mg citicoline and 2000 mg citicoline included both oral and intravenous routes. Consequently, we raise the question of whether the higher incidence of adverse effects with 500 mg citicoline is associated with the differences in administration routes and recommend further investigation of the impact of various administration routes on the efficacy of this drug in the future studies.

## Conclusion

Our research findings revealed that different dosages of citicoline significantly affect the improvement in neurological function, activities of daily living, and the rate of adverse effects in patients with acute IS. Notably, 500 mg citicoline and 2,000 mg citicoline not only demonstrate higher rates of improvement in neurological function and activities of daily living but also have lower mortality and ineffective result. However, due to the varying rankings in the outcome indicators, we did not specify which is the best. Moreover, we also found that 1,000 mg citicoline was not better than 500 mg citicoline and 2,000 mg citicoline in terms of improving neurological function, daily living activities, and reducing mortality. Therefore, we believe that a renewed and comprehensive assessment of 1,000 mg citicoline is warranted. Furthermore, regarding the adverse effect, due to the limited number of original studies involving these three dosages, we consider that there is uncertainty in this aspect of the conclusions, and further exploration is needed after increasing the sample size.

## Data Availability

The original contributions presented in the study are included in the article/Supplementary Material; further inquiries can be directed to the corresponding authors.
