# Supplementary material for: The efficacy of different doses of citicoline in improving the prognosis of patients with acute ischemic stroke based on network meta-analysis
Source: Front Pharmacol. 2025 Apr 4;16:1529647. doi: 10.3389/fphar.2025.1529647 (PMC12006040; doi:10.3389/fphar.2025.1529647)
Supplement: Supplementary file 1 [file DataSheet1.doc]

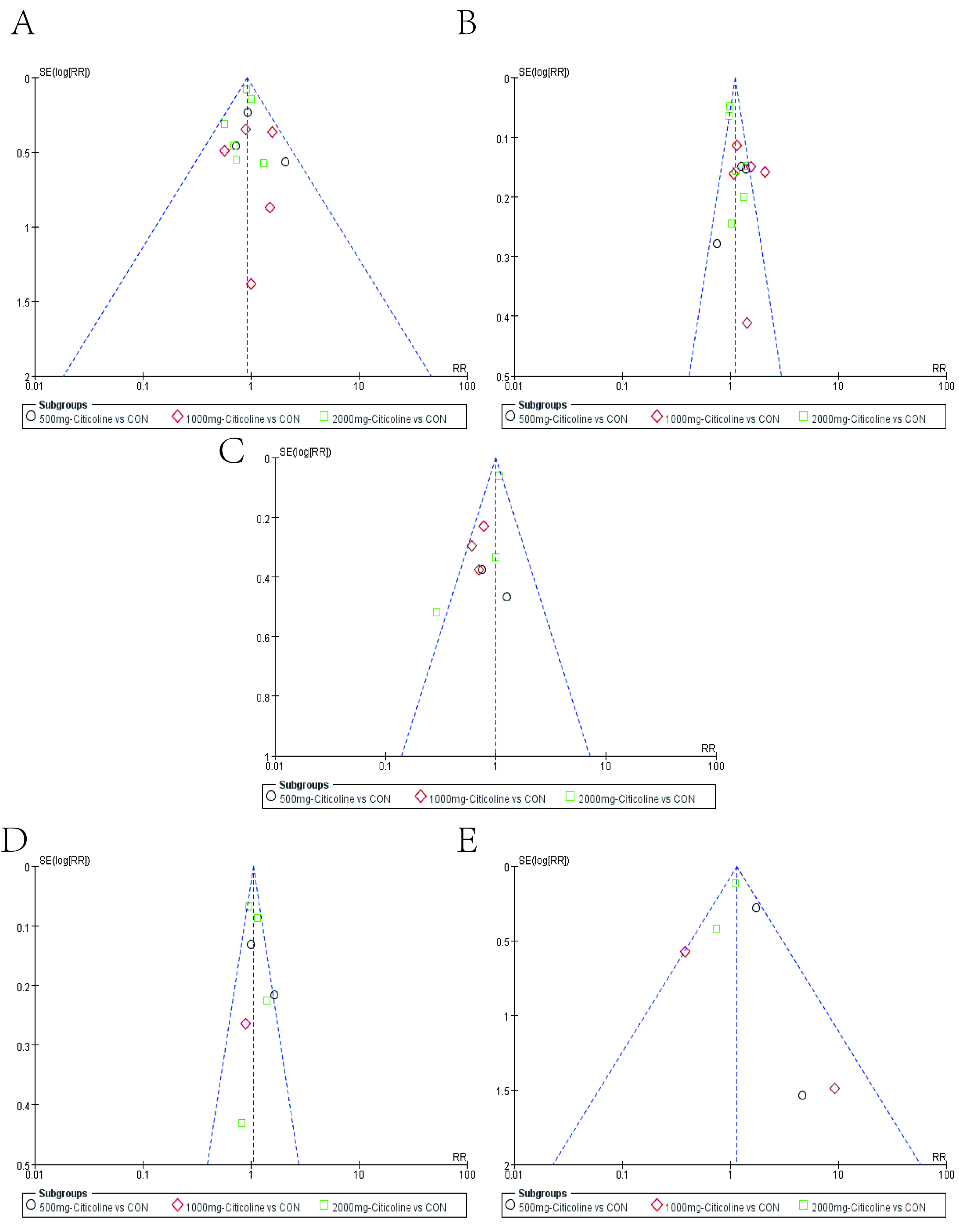


Supplementary Figure 1. The analysis of publication bias

1. The analysis of publication bias in the aspect of death; B. The analysis of publication bias in the aspect of favorable result; C. The analysis of publication bias in the aspect of ineffective result; D. The analysis of publication bias in the aspect of MBI; E. The analysis of publication bias in the aspect of MBI.


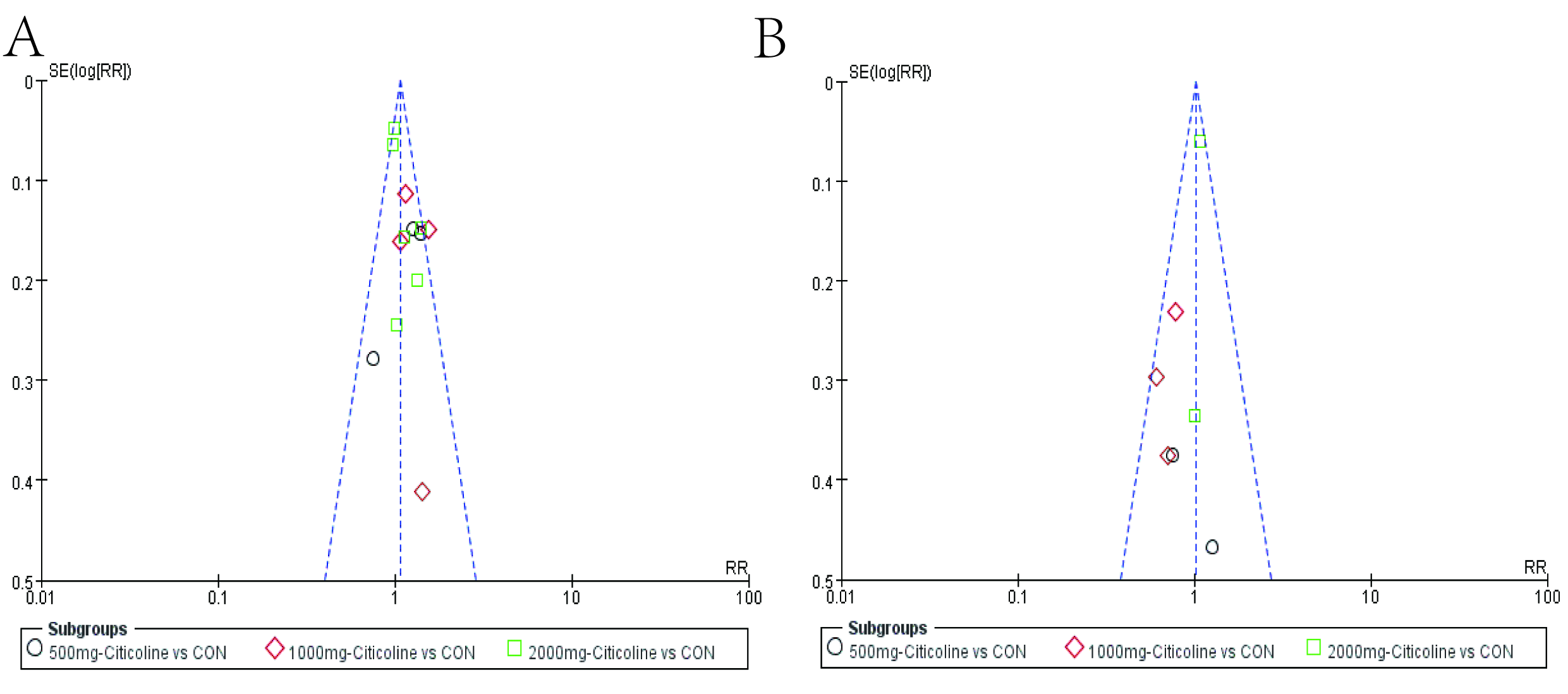


Supplementary Figure 2. The analysis of publication bias after deleting the related-study

1. The analysis of publication bias in the aspect of favorable result after deleting the study of Alvarez-Sabín J, et al. B. The analysis of publication bias in the aspect of ineffective result after deleting the study of Martynov Mlu, et al,.


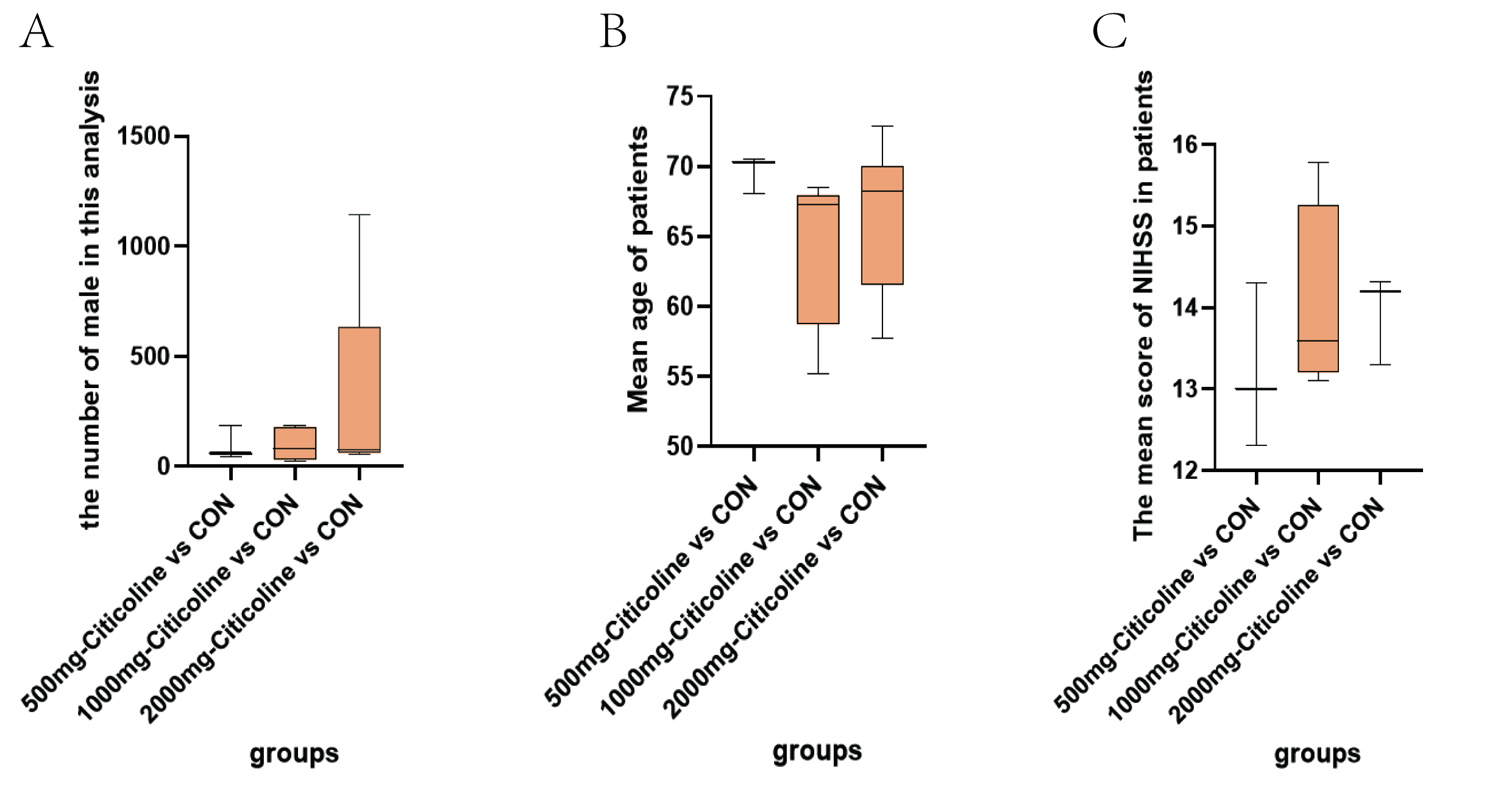


Supplementary Figure 3. The analysis of transitivity

1. The analysis of transitivity in the aspect of the number of male patients; B. The analysis of transitivity in the aspect of the mean age of patients; C. The analysis of transitivity in the aspect of the mean score on NIHSS of patients
