# Supplementary material for: The efficacy of different doses of citicoline in improving the prognosis of patients with acute ischemic stroke based on network meta-analysis
Source: Front Pharmacol. 2025 Apr 4;16:1529647. doi: 10.3389/fphar.2025.1529647 (PMC12006040; doi:10.3389/fphar.2025.1529647)
Supplement: Supplementary file 2 [file DataSheet2.doc]

Supplementary Table 1. The baseline characteristics of the involved study

| Study | Country | Publication year | Study Type | Age(y) | Male | Intervention  Control | Time from stroke onset to treatment | NIHSS | Cases | Outcomes | Follow-up time | Treatment details |
| --- | --- | --- | --- | --- | --- | --- | --- | --- | --- | --- | --- | --- |
| Dávalos A | Spain | 2012 | Multicenter | 72.9±11.8  72.8±12.1 | 588  554 | 2000mg-Citicoline  CON | 4.0-12.3 h  4.0-12.3 h | 11-19  11-19 | 1148  1150 | Death, effective result, adverse result | 90 days | Citicoline 2000mg every day;  Conventional treatment: comprised oxygen therapy, sedation, antiplatelet aggregation, and other treatment measures that decrease blood pressure and intracranial pressure, promote brain cell metabolism and blood circulation to remove blood stasis, and manage water and electrolyte imbalance. |
| Alvarez-Sabín J | USA | 2013 | Single institution | 66.9±11.1  67.7±11.6 | 92  94 | 1000mg-Citicoline  CON | Within 24h  Within 24h | 10-17  10-16 | 172  175 | Death, effective result, adverse result | 6 months | Citicoline 1000 mg every day;  Conventional treatment: comprised oxygen therapy, sedation, antiplatelet aggregation, and other treatment measures that decrease blood pressure and intracranial pressure, promote brain cell metabolism and blood circulation to remove blood stasis, and manage water and electrolyte imbalance. |
| Clark WM | USA | 2001 | Multicenter | 68  67 | 50  54 | 2000mg-Citicoline  CON | 13.2h  13.3h | 13.9  14.5 | 453  446 | Death, effective result, adverse result | 90 days | Citicoline 2000 mg every day;  Conventional treatment: comprised oxygen therapy, sedation, antiplatelet aggregation, and other treatment measures that decrease blood pressure and intracranial pressure, promote brain cell metabolism and blood circulation to remove blood stasis, and manage water and electrolyte imbalance. |
| Warach S | USA | 2000 | Multicenter | 68.5  72.1 | 23  19 | 500mg-Citicoline  CON | 15.1±0.9h  13.5±1.0h | 11.5±0.7  12.7±0.9 | 41  40 | Death, effective result, adverse result | 90 days | Citicoline 500 mg every day;  Conventional treatment: comprised oxygen therapy, sedation, antiplatelet aggregation, and other treatment measures that decrease blood pressure and intracranial pressure, promote brain cell metabolism and blood circulation to remove blood stasis, and manage water and electrolyte imbalance. |
| Clark WM | USA | 1999 | Multicenter | 70  71 | 123  62 | 500mg-Citicoline  CON | 11.7 h  12.3h | 13.3  12.7 | 267  127 | Death, effective result, adverse result | 90 days | Citicoline 500 mg every day;  Conventional treatment: comprised oxygen therapy, sedation, antiplatelet aggregation, and other treatment measures that decrease blood pressure and intracranial pressure, promote brain cell metabolism and blood circulation to remove blood stasis, and manage water and electrolyte imbalance. |
| Mitta M | India | 2012 | Single institution | 54.83±14.46  55.6±14.59 | 13  16 | 1000mg-Citicoline  CON | Within 24h  Within 24h | 15.58±11.36  16±9.23 | 24  25 | Death, effective result | 90 days | Citicoline 1000mg every day;  Conventional treatment: comprised oxygen therapy, sedation, antiplatelet aggregation, and other treatment measures that decrease blood pressure and intracranial pressure, promote brain cell metabolism and blood circulation to remove blood stasis, and manage water and electrolyte imbalance. |
| Alvarez-Sabín J | Spain | 2016 | Single institution | 68.5±9.8  66.4±11.4 | 39  41 | 1000mg-Citicoline  CON | Within 24h  Within 24h | 13  14 | 86  87 | Effective result | 2 years | Citicoline 1000mg every day;  Conventional treatment: comprised oxygen therapy, sedation, antiplatelet aggregation, and other treatment measures that decrease blood pressure and intracranial pressure, promote brain cell metabolism and blood circulation to remove blood stasis, and manage water and electrolyte imbalance. |
| Martynov MIu | Russia | 2012 | Single institution | Male 64.5±11.5  Female 61.0±12.8 | Total 65 | 2000mg-Citicoline  CON | Within 24h  Within 24h | NR  NR | 89  52 | Death, effective result | NR | Citicoline 2000mg every day;  Conventional treatment: comprised oxygen therapy, sedation, antiplatelet aggregation, and other treatment measures that decrease blood pressure and intracranial pressure, promote brain cell metabolism and blood circulation to remove blood stasis, and manage water and electrolyte imbalance. |
| Clark WM | USA | 1997 | Multicenter | 66  67  68  70 | 30  35  27  29 | 500mg-Citicoline  1000mg-Citicoline  2000mg-Citicoline  CON | 14.7h  14.6h  14.6h  14.2h | 11.6  13.2  13.6  13.0 | 62  66  66  65 | Death, effective result | 90 days | Citicoline 500mg/ 1000mg/ 2000mg every day;  Conventional treatment: comprised oxygen therapy, sedation, antiplatelet aggregation, and other treatment measures that decrease blood pressure and intracranial pressure, promote brain cell metabolism and blood circulation to remove blood stasis, and manage water and electrolyte imbalance. |
| Leon-Jimenez C | Mexico | 2010 | Multicenter | 68.6  69.6 | 42  41 | 2000mg-Citicoline  CON | Within 24h  Within 24h | 14.3  14.33 | 86  87 | Death, effective result | 90 days | Citicoline 2000mg every day;  Conventional treatment: comprised oxygen therapy, sedation, antiplatelet aggregation, and other treatment measures that decrease blood pressure and intracranial pressure, promote brain cell metabolism and blood circulation to remove blood stasis, and manage water and electrolyte imbalance. |
| Tazaki Y | Japan | 1988 | Single intitution | 29-90  29-90 | 87  93 | 1000mg-Citicoline  CON | Within 24h  Within 24h | NR  NR | 131  136 | Death, effective result, adverse result | NR | Citicoline 1000mg every day;  Conventional treatment: comprised oxygen therapy, sedation, antiplatelet aggregation, and other treatment measures that decrease blood pressure and intracranial pressure, promote brain cell metabolism and blood circulation to remove blood stasis, and manage water and electrolyte imbalance. |
| Mehta A | India | 2019 | Single institution | 59.5  64.9 | 12  11 | 1000mg-Citicoline  CON | 13.47±4.34h  13.30±4.95h | 14±4.34  13.35±4.53 | 20  20 | Death, adverse result | 90 days | Citicoline 1000mg every day;  Conventional treatment: comprised oxygen therapy, sedation, antiplatelet aggregation, and other treatment measures that decrease blood pressure and intracranial pressure, promote brain cell metabolism and blood circulation to remove blood stasis, and manage water and electrolyte imbalance. |
| Agarwal A | India | 2022 | Single institution | 61±14.5  54.5±14.6 | 30  30 | 2000mg-Citicoline  CON | Within 24h  Within 24h | 8-42  8-42 | 49  40 | Death, effective result, adverse result | 90 days | Citicoline 2000mg every day;  Conventional treatment: comprised oxygen therapy, sedation, antiplatelet aggregation, and other treatment measures that decrease blood pressure and intracranial pressure, promote brain cell metabolism and blood circulation to remove blood stasis, and manage water and electrolyte imbalance. |

* y: year; CON: conventional treatment; h: hours; NR: Not report

Supplementary Table 2. The evaluation of the global inconsistency

| Outcome measures | Chi2 | *P* |
| --- | --- | --- |
| Death | 3.67 | 0.0554 |
| Favorable result | 2.29 | 0.5143 |
| Ineffective effect | 0.72 | 0.8690 |
| MBI | 3.77 | 0.1521 |

Supplementary Table 3. The local inconsistency

Supplementary Table 3a. The local inconsistency of the death

| Loop | IF | Z_value | P | 95%CI | τ^2^ |
| --- | --- | --- | --- | --- | --- |
| 1000mg Citicoline-500mg Citicoline-CON | 1.201 | 1.916 | 0.055 | (0.00,2.43) | 0.000 |

Supplementary Table 3b. The local inconsistency of the favorable result

| Loop | IF | Z_value | P | 95%CI | τ^2^ |
| --- | --- | --- | --- | --- | --- |
| 1000mg Citicoline-500mg Citicoline-CON | 0.962 | 1.219 | 0.223 | (0.00,2.51) | 0.254 |
| 1000mg Citicoline-2000mg Citicoline-CON | 0.610 | 0.930 | 0.352 | (0.00,1.89) | 0.111 |
| 2000mg Citicoline-500mg Citicoline-CON | 0.109 | 0.183 | 0.855 | (0.00,1.28) | 0.057 |

Supplementary Table 3c. The local inconsistency of the ineffective result

| Loop | IF | Z_value | P | 95%CI | τ^2^ |
| --- | --- | --- | --- | --- | --- |
| 1000mg Citicoline-500mg Citicoline-CON | 0.485 | 0.469 | 0.469 | (0.00,1.80) | 0.000 |
| 2000mg Citicoline-500mg Citicoline-CON | 0.575 | 0.361 | 0.718 | (0.00,1.33) | 0.000 |
| 1000mg Citicoline-2000mg Citicoline-CON | 0.005 | 0.006 | 0.996 | (0.00,1.72) | 0.173 |

Supplementary Table 3d. The local inconsistency of the MBI

| Loop | IF | Z_value | P | 95%CI | τ^2^ |
| --- | --- | --- | --- | --- | --- |
| 1000mg Citicoline-500mg Citicoline-CON | 0.872 | 1.516 | 0.130 | (0.00,2.00) | 0.000 |
| 1000mg Citicoline-2000mg Citicoline-CON | 0.537 | 1.005 | 0.315 | (0.00,1.58) | 0.000 |
| 2000mg Citicoline-500mg Citicoline-CON | 0.055 | 0.097 | 0.923 | (0.00,1.16) | 0.046 |

Supplementary Table 4. The evaluation of inconsistency

Supplementary Table 4a. The evaluation of inconsistency of the death

| Comparisons | Direct | | Indirect | | | Difference | |
| --- | --- | --- | --- | --- | --- | --- | --- |
|  | SMD | SE | SMD | SE | SMD | SE | *P* |
| 1000mg-Citicoline vs CON | 0.22 | 0.29 | -0.99 | 0.56 | 1.20 | 0.63 | 0.055 |
| 1000mg-Citicoline vs 500mg-Citicoline | -0.92 | 0.49 | 0.28 | 0.39 | -1.20 | 0.63 | 0.055 |
| 2000mg-Citicoline vs CON | ... | ... | ... | ... | ... | ... | ... |
| 500mg-Citicoline vs CON | -0.06 | 0.26 | 1.14 | 0.57 | -1.20 | 0.63 | 0.055 |

Supplementary Table 4b. The evaluation of inconsistency of the favorable result

| Comparisons | Direct | | Indirect | | | Difference | |
| --- | --- | --- | --- | --- | --- | --- | --- |
|  | SMD | SE | SMD | SE | SMD | SE | *P* |
| 1000mg-Citicoline vs 2000mg-Citicoline | 0.13 | 0.53 | -0.58 | 0.33 | 0.70 | 0.63 | 0.263 |
| 1000mg-Citicoline vs 500mg-Citicoline | 0.43 | 0.53 | -0.65 | 0.44 | 1.08 | 0.69 | 0.115 |
| 1000mg-Citicoline vs CON* | -0.68 | 0.24 | 0.26 | 0.87 | -0.94 | 0.90 | 0.297 |
| 2000mg-Citicoline vs 500mg-Citicoline | 0.29 | 0.58 | 0.05 | 0.45 | 0.24 | 0.73 | 0.743 |
| 2000mg-Citicoline vs CON* | -0.22 | 0.21 | -0.68 | 0.91 | 0.46 | 0.94 | 0.623 |
| 500mg-Citicoline vs CON* | -0.29 | 0.32 | -1.12 | 0.86 | 0.83 | 0.92 | 0.367 |

* all the evidence about these contrasts comes from the trials which directly compare them

| Comparisons | Direct | | Indirect | | | Difference | |
| --- | --- | --- | --- | --- | --- | --- | --- |
|  | SMD | SE | SMD | SE | SMD | SE | P |
| 1000mg-Citicoline vs CON* | 0.56 | 0.34 | 0.38 | 1.08 | 0.19 | 1.13 | 0.867 |
| 1000mg-Citicoline vs 2000mg-Citicoline | 0.45 | 0.61 | 0.32 | 0.62 | 0.12 | 0.87 | 0.885 |
| 1000mg-Citicoline vs 500mg-Citicoline | 0.07 | 0.56 | 0.80 | 0.66 | -0.72 | 0.86 | 0.403 |
| 2000mg-Citicoline vs CON* | 0.18 | 0.35 | -0.23 | 1.11 | 0.41 | 1.16 | 0.724 |
| 2000mg-Citicoline vs 500mg-Citicoline | -0.35 | 0.61 | 0.44 | 0.76 | -0.77 | 0.98 | 0.422 |
| 500mg-Citicoline vs CON* | 0.06 | 0.44 | 0.81 | 1.06 | -0.74 | 1.13 | 0.512 |

Supplementary Table 4c. The evaluation of inconsistency of the ineffective effect

* all the evidence about these contrasts comes from the trials which directly compare them

| Comparisons | Direct | | Indirect | | | Difference | |
| --- | --- | --- | --- | --- | --- | --- | --- |
|  | SMD | SE | SMD | SE | SMD | SE | P |
| 1000mg-Citicoline vs 2000mg-Citicoline* | 0.72 | 0.44 | 0.26 | 0.79 | 0.47 | 0.89 | 0.600 |
| 1000mg-Citicoline vs 500mg-Citicoline* | 1.03 | 0.40 | -0.22 | 0.79 | 1.25 | 0.88 | 0.154 |
| 1000mg-Citicoline vs CON* | 0.17 | 0.40 | 1.47 | 0.60 | -1.30 | 0.72 | 0.068 |
| 2000mg-Citicoline vs 500mg-Citicoline* | 0.30 | 0.43 | 0.10 | 0.36 | 0.20 | 0.56 | 0.718 |
| 2000mg-Citicoline vs CON* | -0.09 | 0.13 | 1.16 | 0.87 | -1.25 | 0.88 | 0.154 |
| 500mg-Citicoline vs CON* | 0.31 | 0.28 | 0.16 | 0.83 | -0.47 | 0.89 | 0.600 |

Supplementary Table 4d. The evaluation of inconsistency of the MBI

* all the evidence about these contrasts comes from the trials which directly compare them
